# Influence of Tryptophan and Serotonin on Mood and Cognition with a Possible Role of the Gut-Brain Axis

**DOI:** 10.3390/nu8010056

**Published:** 2016-01-20

**Authors:** Trisha A. Jenkins, Jason C. D. Nguyen, Kate E. Polglaze, Paul P. Bertrand

**Affiliations:** 1 School of Medical Sciences, Health Innovations Research Institute, RMIT University, Melbourne, Victoria 3083, Australia; jason.nguyen2@student.rmit.edu.au (J.C.D.N.); kate.polglaze@rmit.edu.au (K.E.P.); paul.bertrand@rmit.edu.au (P.P.B.); 2 School of Medical Sciences, University of New South Wales, Sydney 2052, Australia

**Keywords:** gut-brain axis, mood and cognition, tryptophan, serotonin

## Abstract

The serotonergic system forms a diffuse network within the central nervous system and plays a significant role in the regulation of mood and cognition. Manipulation of tryptophan levels, acutely or chronically, by depletion or supplementation, is an experimental procedure for modifying peripheral and central serotonin levels. These studies have allowed us to establish the role of serotonin in higher order brain function in both preclinical and clinical situations and have precipitated the finding that low brain serotonin levels are associated with poor memory and depressed mood. The gut-brain axis is a bi-directional system between the brain and gastrointestinal tract, linking emotional and cognitive centres of the brain with peripheral functioning of the digestive tract. An influence of gut microbiota on behaviour is becoming increasingly evident, as is the extension to tryptophan and serotonin, producing a possibility that alterations in the gut may be important in the pathophysiology of human central nervous system disorders. In this review we will discuss the effect of manipulating tryptophan on mood and cognition, and discuss a possible influence of the gut-brain axis.

## 1. Introduction

Tryptophan is an essential amino acid found in many protein-based foods and dietary proteins [1] including meats, dairy, fruits, and seeds. High-glycaemic index and -glycaemic load meals also increase the availability of tryptophan [2]. Levels of plasma tryptophan are determined by a balance between dietary intake [3], and its removal from the plasma as a part of its essential role in protein biosynthesis [4]. Aside from its role in protein formation, tryptophan is a precursor for a number of metabolites, most notably kynurenine and the neurotransmitter, serotonin which is the focus of this review.

## 2. Serotonin and Kynurenine

Tryptophan is the sole precursor of peripherally and centrally produced serotonin [4]. However, the second most prevalent metabolic pathway of tryptophan after protein synthesis is the synthesis of kynurenine, which accounts for approximately 90% of tryptophan metabolism [5]. Kynurenine is the precursor of kynurenic acid, an antagonist at glutamate ionotropic receptors. There is strong evidence implicating the kynurenines in behavioural and cognitive symptoms of neurological disease [6], however the relationship between the central effects of tryptophan depletion/supplementation and the kynurenine pathway is as yet not clear [7,8,9]. The role of kynurenine in the brain is beyond the scope of this review.

### Serotonin and Its Receptors

Serotonin synthesis occurs in the periphery within the gut neurons and enterochromaffin cells and centrally within the neurons of the raphe in the brain stem. The effects of tryptophan depletion on peripheral serotonin production will be discussed later. For central serotonin production to occur, tryptophan first needs to gain access to the central nervous system (CNS) via the blood-brain barrier. Tryptophan is a substrate for the large neutral amino-acid transporter system and competes for transport with several other amino acids essential for brain function. This competition for transport is the basis for some acute tryptophan depletion diets (e.g., [10]). It is generally accepted that most of our tryptophan is bound to plasma albumin and hence is unavailable for transport into the brain. This normally limits the tryptophan available for central serotonin synthesis but release of tryptophan from this pool could increase transport. In addition to free tryptophan levels, findings from exercise studies demonstrate that there must be other, currently unknown, mechanisms controlling central uptake of tryptophan [11]. Once in the CNS, l-tryptophan is hydroxylated to 5-hydroxytryptophan by the enzyme tryptophan hydroxylase type 2, the rate limiting step in brain serotonin synthesis. This is followed by subsequent decarboxylation involving the enzyme l-aromatic acid decarboxylase to serotonin (5-hydroxytryptamine, 5-HT). Serotonin is then taken up into vesicles by the vesicular monoamine transporter isoform 2 of the raphe neurons. Degradation of serotonin is via monoamine oxidase type A and aldehyde dehydrogenase to the major serotonin metabolite 5-hydroxyindoleacetic acid (5HIAA). Levels of serotonin are also influenced by the tryptophan-degrading enzyme, indoleamine 2,3-dioxygenase and tetrahydrobiopterin, the cofactor of tryptophan hydroxylase.

All but one subtype of the many serotonin receptors are metabotropic G protein–coupled receptors. Multiple serotonin receptors have been found, with receptor families from 5-HT_1_ to 5-HT_7_ [12,13]. The 5-HT_3_ receptor is unique among the currently known serotonergic receptor subtypes in that it belongs to the ionotropic, ligand-gated ion channel family. Serotonergic neurons innervate large areas of the human brain, with most projections arising from neuronal cell bodies in the dorsal and median raphe and neighbouring nuclei of the lower brain stem. There are projections to the hippocampus, amygdala, hypothalamus, thalamus, neocortex, and basal ganglia, although most structures receive some serotonergic innervation [14]. Through this diffuse network within the central nervous system, serotonin modulates a wide array of functions including sleep, control of appetite and temperature, and the focus of this review, mood and cognition.

## 3. Serotonin and Mood

Lowered mood is one of the major symptoms of depression, an affective disorder which is the leading cause of disability worldwide, affecting approximately 20% of the world’s population [15]. The major therapeutic agents for treating depression are antidepressants, mostly selective serotonin reuptake inhibitors or combined serotonin/noradrenaline reuptake inhibitors [16]. The mechanism of these medications is believed to be in part by increasing synaptic levels of monoamines, mainly serotonin and noradrenaline and subsequent activation of serotoninergic and noradrenergic postsynaptic and autoreceptors [17]. The therapeutic benefits of increased levels of monoamines were discovered in the middle of last century, when monoamine oxidase inhibitors and tricyclic antidepressants showed efficacy in treating depression. This led to the monoamine hypothesis where depression was thought to be caused by a deficiency in monoamine neurotransmitters [18]. However, antidepressants are only partly effective in the treatment of depression of moderate and greater severity in adults (response rates of approximately 48% compared with 30% for placebo) [19,20], suggesting that the monoamine hypothesis only partially explains depression [21,22].

The effect of serotonin on mood has been investigated using an acute tryptophan depletion technique where lowering dietary tryptophan levels causes a lowering of brain serotonin levels, allowing analysis of serotonin-dependent behaviour [23]. This is discussed in more detail below.

## 4. Serotonin and Cognition

The serotonergic system plays a role in behaviours that involve a high cognitive demand. Serotonin receptors are found in brain regions involved in learning and memory including the cortex, amygdala, and hippocampus [24]. As drug targets for cognitive improvement or enhancement, serotonin receptors have received attention with a focus on several serotonin-receptor subtypes shown to be involved in cognition and memory. Converging evidence suggests that the administration of 5-HT_2A/2C_ or 5-HT_4_ receptor agonists or 5-HT_1A_ or 5HT_3_ and 5-HT_1B_ receptor antagonists prevents memory impairment and facilitates learning in situations involving a high cognitive demand. In contrast, receptor antagonists for 5-HT_2A/2C_ and 5-HT_4_, or agonists for 5-HT_1A_ or 5-HT_3_ and 5-HT_1B_ generally have opposite effects on memory and learning [25,26,27,28,29,30].

Whether serotonin plays a role in modulating cognitive function through specific effects on learning, memory and executive function are still not understood. This may be attributed partially to the differing roles of various serotonin receptor subtypes in cognition [30]. However, lowering central serotonin levels through tryptophan depletion experimentally has enabled some elucidation of the role of serotonin in different modes of learning.

## 5. Tryptophan Depletion

Initial studies aiming to deplete central tryptophan employed the irreversible tryptophan hydroxylase inhibitor, 4-chloro-dl-phenylalanine methyl ester (PCPA), which depletes serotonin by stopping the rate-limiting step in its synthesis [31]. However concerns about its toxicity and dose range largely limited its experimental use [23].

An alternative to inhibiting the synthesis enzyme for serotonin is to deplete its substrate tryptophan from the brain. Rapid dietary depletion of tryptophan allows the investigation of the effect of lowered tryptophan levels, and as such provides a paradigm for studying the role of serotonin in central processes. The ingestion of a diet or solution containing large neutral amino acids but deficient in tryptophan induces an acute and reliable lowering of plasma tryptophan. This effect is thought to be due to the phenomena that removal of tryptophan from the diet stimulates protein synthesis in the liver, which uses up the available plasma tryptophan. This effect has been observed experimentally in animals including mice [32], rats [33,34,35], and primates [36]; and in humans [37,38].

In addition to increased liver protein synthesis, the large neutral amino acids included in the diet compete with tryptophan for transport across the blood brain barrier and thus restrict the entry of tryptophan into the brain. This depletes tryptophan, and thus serotonin, centrally. Rodent studies have shown that acute tryptophan depletion reduced tryptophan levels in the brain by up to 70% [35], with associated decreases in central serotonin and reduced 5-HT_1A_ receptor binding [35,39]. In humans acute tryptophan depletion inhibits serotonin synthesis [40] and also lowers cerebrospinal fluid concentrations of tryptophan [41] and 5-hydroxyindoleacetic acid (5-HIAA), the major serotonin metabolite [41,42].

## 6. Tryptophan Depletion, Serotonin and Mood

Clinical and preclinical studies have used the tryptophan depletion model to investigate the idea that low serotonin synthesis is associated with depressed mood [43,44].

### 6.1. Clinical Studies

Tryptophan depletion studies in never-depressed individuals are variable, with no or little overall effect on lowering of mood [45,46]. Interestingly, reports of moderate mood lowering are seen more often in studies with healthy women than in studies with healthy men [47]. However in never-depressed healthy volunteers who are at high risk for depression through a familial risk factor, acute tryptophan depletion produces clear abnormalities in mood control [48,49]. Finally, in remitted depressed patients, temporarily lowering tryptophan levels can result in an acute depressive relapse [50,51,52] with transient exacerbation of symptoms associated with patients taking serotonergic anti-depressants [53,54]. These studies reveal that subjects with a pre-existing vulnerability in the serotonergic system may be most susceptible to a tryptophan challenge. Moreover, low serotonin can indeed contribute to a lowered mood state, however this cannot occur in isolation—it must be in concert with some other unknown system (perhaps neurotransmitter or genetic) that interacts with the reduced serotonin to decrease mood.

### 6.2. Preclinical Studies

Assessing animal models of lowered mood brings us to models of high anxiety, depression, or despair. The phenotypic behaviours that are associated with these models can be quantified and used for investigation of novel therapies for human disease.

Previous studies have shown that low dietary tryptophan seems to have an anxiogenic and depressant effect on rat behaviour. After one month of treatment with a low tryptophan diet, experimental rats displayed a significant increase in immobility counts in the forced swimming test and exhibited anxiety-like behaviour in the elevated plus maze test [55]. Moreover tryptophan-limited mice showed increased defensive aggression in the resident-intruder test and enhanced social dominance in the social dominance tube suggesting that dietary tryptophan restriction appears to result in alterations in the emotional response to stress [56]. Unfortunately, the results of acute dietary tryptophan manipulation appear to be species and strain specific. For example, in mice, acute tryptophan depletion did not show anxiety in the elevated zero-maze and did not show increased immobility in the forced swimming test or tail suspension test even though the manipulation resulted in a 74% reduction of plasma tryptophan [57]. Similarly, in a rat study comparing acute tryptophan depletion effects between Brown Norway and Sprague Dawley rats, Sprague Dawley rats showed more anxiety- and depression-related behaviour compared to the non-albino strain, even with a 60% decrease in plasma tryptophan observed in both strains [34]. These results suggest that acute tryptophan depletion effects are likely to be strain dependent on the behavioural and the neurochemical level.

## 7. Tryptophan Depletion, Serotonin and Cognition

Tryptophan depletion studies have also been performed in clinical and preclinical studies to assess the relationship between a lowered serotonin system and cognition [30].

### 7.1. Clinical Studies

A comprehensive meta-analysis of over fifty tryptophan depletion studies in man from 1966 through to 2008 was published by Mendelsohn and colleagues in 2009 [58]. The effects of acute tryptophan depletion on psychomotor processing, declarative memory, working memory, executive functions, and attention were evaluated with the most robust finding that lowering tryptophan impaired the consolidation of episodic memory for verbal information [38,59]. Semantic memory appeared to be unaffected by acute tryptophan depletion as were verbal, spatial, and affective working memory, executive function, and attention [58].

Many of the studies covered in the aforementioned review [58] focus on healthy volunteers, or those with susceptibility to depression. Latter work published after Mendelsohn’s review has demonstrated some interesting findings concerning emotional processing. In a small study of depressed patients, a bimodal symptom response to acute tryptophan depletion was shown to be preceded by a bimodal emotional processing bias in the same direction; that is, patients whose depressive symptoms improved 24 h after depletion showed more positive emotional processing bias 5 h after depletion, while the reverse was true for patients whose mood symptoms worsened [60]. Asymptomatic individuals at high familial risk for depression also showed abnormalities in emotional processing while undergoing acute tryptophan depletion [48]. Interestingly in normal subjects, acute tryptophan depletion elicited significantly lower intensity and arousal ratings for angry faces in an unconscious perception task [61]. In another study involving tryptophan depletion in postmenopausal women, there was an increase in brain activation in the orbital frontal cortex and bilateral amygdala, as measured by functional magnetic resonance imaging, during an emotional processing task as compared to untreated controls [62].

Manipulating central tryptophan levels using acute tryptophan depletion is also used as a tool to investigate the role of serotonin in neurological disorders. In Parkinson’s disease patients, a demonstrable reduction in global cognitive function and verbal recognition during acute tryptophan depletion is observed compared with placebo and control patients suggesting an interaction between serotonergic and cholinergic impairment [63]. No deficits in memory were observed in tryptophan-depleted young persons with attention deficit hyperactivity disorder [64], in reward response tests with alcoholic males [65], or in cognitive testing of Alzheimer’s patients that could not be attributed to old age [66]. Interestingly it was observed that the detrimental effects of acute tryptophan depletion on working memory were more common in an elderly, compared to young, group of healthy volunteers [45].

Manipulating tryptophan levels using acute tryptophan depletion has also been used to investigate the role of serotonin in other disorders. Kennedy and colleagues have used acute tryptophan depletion to demonstrate that impaired hippocampal-mediated cognitive performance in irritable bowel syndrome [67] is modulated by peripheral tryptophan levels [68]. Moreover, in breast cancer survivors, acute tryptophan depletion was used to model serotonin loss which is a common side effect of oestrogen withdrawal in this disease population. This study demonstrated specific impairment in episodic memory and motor speed suggesting a critical role for serotonin in cognitive impairment in these patients [69].

### 7.2. Preclinical Studies

Acute oral administration of a tryptophan-free protein-carbohydrate mixture to rats significantly lowered hippocampus tryptophan levels [70] and produced impaired performance in the visual working memory novel object recognition test [34,71,72,73], but not sustained attention [70] or spatial learning [71,74] tasks. In contrast, chronic tryptophan depletion, which simulates a long term reduction in central serotonin in brain regions including the hippocampus, frontal cortex, and striatum in rodents [75], impaired object-recognition memory [75] and hippocampal-dependent contextual fear memory [76]. In addition, chronic tryptophan dietary restriction enhanced an amphetamine-induced prepulse inhibition impairment [77], confirming previous observations from this research group on the sensitization to other amphetamine-mediated behavioural manifestations induced by a prolonged tryptophan-poor dietary regimen [78].

## 8. Tryptophan Supplementation and Cognition

A strategy of administration of tryptophan-rich dietary proteins can enhance tryptophan availability to the brain and thus potentially model enhanced serotonin synthesis. Clinical studies have found that acute tryptophan supplementation improved serial reaction times and attention scores [79] and abstract visual memory [80], while chronic (14 days) supplementation increased positive facial recognition memory, and decreased baseline startle responsivity [81]. In addition, Rondanelli *et al.* [82] gave a 12-week diet of docosahexaenoic acid phospholipids with melatonin and tryptophan to elderly patients suffering from mild cognitive impairment. They reported significant improvements in several measures of cognitive function including the Mini-Mental State Examination [82], however with this mixed diet it is difficult to make a conclusion about the role of serotonin.

## 9. Tryptophan, Sleep, Mood and Cognition

Tryptophan has been shown to have direct effects on sleep, producing an increase in rated subjective sleepiness, and decrease in total wakefulness [83,84]. This improved quality of sleep is associated with an improvement in hedonic and cognitive measures [79], improved morning alertness and brain measures of attention [85].

Acute tryptophan depletion studies in humans demonstrate inhibition of rapid eye movement (REM) latency and prolonged REM sleep [86,87], with further work from animal studies demonstrating the importance of serotonin in this association [88]. Serotonin is also a precursor to melatonin in the pineal gland.

Patients with depression suffer from poor sleep quality [89], with associated antidepressant treatment often exacerbating sleep inefficiency with insomnia and decreased total sleep time being common side-effects [90]. The effect of tryptophan depletion on sleep in depression has largely focused on remitted patients-acute tryptophan depletion in these patients, who were still taking antidepressants, resulted in reduced sleep and REM latencies but increased density [91,92], demonstrating that depleting tryptophan did not alter the antidepressant side-effects. Interestingly, in a population of patients with obsessive compulsive disorder, tryptophan depletion induced a worsening of sleep continuity, but no changes of REM or slow wave sleep [93].

## 10. Tryptophan, Serotonin and the Brain-Gut Axis

**Figure 1:**
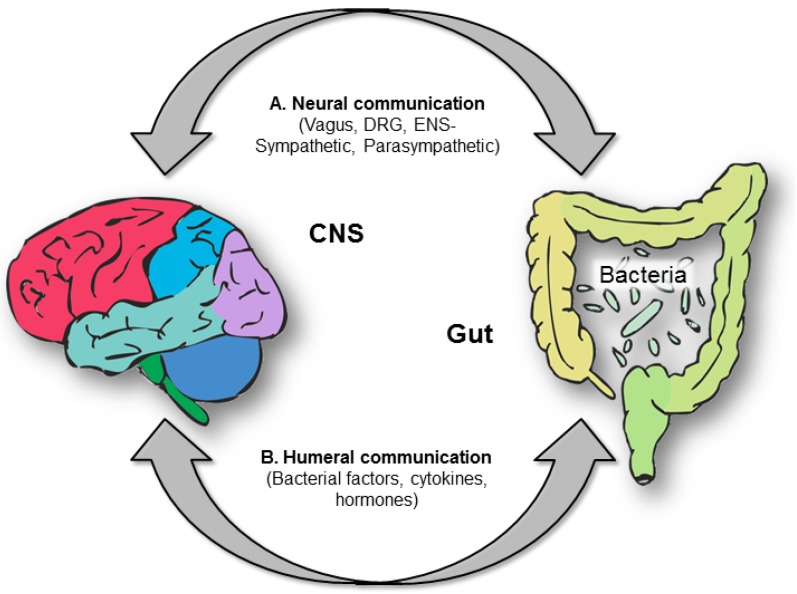
The brain-gut axis and the bi-directional system of communication. The brain-gut axis is a bi-directional system of communication between the brain and the gastrointestinal tract. This links emotional and cognitive centres of the brain with peripheral control and function of the gut and its resident microbiota. Serotonin is a key element of this axis, acting as a neurotransmitter in the CNS and in the enteric nervous system that is present in the wall of the gut. A. Neural communication between the gut and brain is via the vagus (stomach and rectum) and dorsal root ganglia (DRG-small and large intestine), via projections from the enteric nervous system to sympathetic ganglia and parasympathetic innervation of the gut. B. Humeral communication is via release of bacterial factors, production of cytokines and circulating hormones. An important advance for future studies will be testable models of a potential mechanism of action (e.g., cutting the vagus can block some effects of changing the gut microbiota in rodent models).

The brain-gut axis is a bi-directional system of communication between the brain and the gastrointestinal tract, linking emotional and cognitive centres of the brain with peripheral control and function of the gut (Figure 1). Serotonin is a key element of this axis, acting as a neurotransmitter in the CNS and in the enteric nervous system that is present in the wall of the gut. In addition, serotonin is produced by endocrine cells and acts as a paracrine hormone in the gut and as an endocrine hormone, carried through the blood bound to platelets. Its role as a hormone acts to link the two ends of the brain-gut axis as well as having systemic effects such as bone density and metabolism [94,95]. Central serotonin production represents just 5% of total serotonin synthesis, with the vast majority of serotonin made in the periphery. Peripheral synthesis occurs in tissues such as bone, mammary glands, the pancreas, but the gastrointestinal epithelium is by far the largest source. The enterochromaffin cells in the gastrointestinal epithelium account for ~90% of all serotonin synthesis. The peripheral endocrine synthesis pathway only differs from the central and enteric neuronal pathways by the utilisation of tryptophan hydroxylase type 1 instead of type 2 [96,97]. Degradation of serotonin is via monoamine oxidase and aldehyde dehydrogenase to 5HIAA as in the CNS, but in the periphery glucuronidation also plays an important role [98].

### 10.1. Tryptophan and the Gut Microbiota

Another piece of the serotonin puzzle involves the resident community of microorganisms that have colonised the digestive tract. The gut microbiota is primarily found in the large intestine, but smaller numbers can be found throughout the gastrointestinal tract [99]. Cross-talk between the gastrointestinal epithelium and enteric flora contributes to functions such as immune responses and regulation of hormones, and is proving to be critical to the maintenance of both homeostasis and health (Figure 1). How the bacterial community establishes early in life [100], or changes across the lifespan, can have consequences on the metabolism of tryptophan, and thus the serotonergic system. A balance is needed between bacterial utilization of tryptophan and the tryptophan necessary for serotonin synthesis in both enteric and central nervous systems [101].

There is both direct and indirect regulation of tryptophan and serotonin in the gut by the resident microbiota. Indirect regulation of tryptophan availability and serotonin formation by the gut microbiota is primarily via the kynurenine pathway. As noted, the synthesis of kynurenine accounts for approximately 90% of tryptophan metabolism [5]. Recent evidence for direct regulation comes from germ-free animals that are laboratory-raised and are gut microbiota-deficient. These animals show increased levels of circulating tryptophan [102] and decreased serotonin [103]. When these animals have tryptophan metabolising bacteria introduced to their gut, circulating levels of tryptophan fall, with this alteration accompanying a sex-specific effect on hippocampal serotonin concentrations in male germ-free animals [102]. Within the brain, an increase in hippocampal serotonin levels and turnover was observed, along with a decrease in anxiety-like behaviour, demonstrating the influence of gut microbiota on both behavioural correlates and brain neurochemistry [104]. Interestingly, these animals also displayed a reduction in brain-derived neurotrophic factor messenger RNA levels and reduced expression of the synaptic signalling genes PSD-95 and synaptophysin in regions of the brain responsible for motor control and anxiety such as the striatum [104].

In irritable bowel syndrome, changes in the balance of microbiota are associated with symptomatology as well as alterations to both gut and brain serotonin levels [105,106]. Moreover, the expression of toll-like receptors, which act to alert the body to pathogens, are altered in both plasma and colonic samples from irritable bowel syndrome patients [107,108]. Recent data also shows that bacterial products such as short chain fatty acids can upregulate serotonin production by the enterochromaffin cells [109].

### 10.2. Behaviour and the Gut Microbiome

As discussed, central serotonin plays a major role in mood and cognition. An influence of gut microbiota on behaviour is becoming increasingly evident, via a variety of proposed mechanisms including changes to tryptophan uptake and serotonin synthesis.

Germ-free mice display less anxiety-like behaviours than their traditionally colonised counterparts [102,110]. Meanwhile, chronic treatment with lactic acid bacteria *Lactobacillus rhamnosus* to mice induced alterations in GABA receptors in cortical hippocampus, and amygdala in comparison with control-fed mice, while also reducing stress-induced corticosterone levels and anxiety- and depression-related behaviour [111]. Interestingly, these effects were not found in vagotomized mice, identifying the vagus as a major modulatory communication pathway between the gut bacteria and the brain [111] (Figure 1A).

In animal models of depression, both environmental [112] and surgical [113], animals display depressive-like behaviour and an altered intestinal microbial profile. These findings have now been replicated within a clinical population. In a recent study in major depression patients, several predominant genera were found in significantly different levels between the depressive and control groups showing either a predominance of some potentially harmful bacterial groups or a reduction in beneficial bacterial genera [114].

The influence of the gut microbiota on behaviour also extends to cognitive function in preclinical models, though all animal behavioural testing has an anxiety component, this suggests that cognitive deficits are not observed without a degree of stress. Mice infected with an enteric pathogen exhibited working memory dysfunction [115], and socially-associated behavioural impairment [116] but only after acute water stress. Clinically, there is discussion of the engagement of gut microbial flora in the pathogenesis of Alzheimer’s disease, but at this stage this is speculative [117,118].

### 10.3. Tryptophan Depletion and the Gut-Brain Axis

The central control of pain is an important component of irritable bowel syndrome and serotonin has been shown to play a role. In healthy women, a painful balloon distension to the rectum resulted in increased brain activity as shown by functional magnetic resonance imaging. When these stimuli were repeated during acute tryptophan depletion, there was an enhanced response in the amygdala, emotional arousal areas, and homeostatic afferent networks. There was also a decrease in negative feedback inhibition of the amygdala. When these tests were repeated in women with constipation-predominant irritable bowel syndrome, a similar pattern of brain activity was observed. This suggests that there are enhanced change in brain activity, namely the homeostatic afferent network and the emotional arousal network, after aversive visceral stimulation [119,120].

In addition, cognitive performance is altered in irritable bowel syndrome [121]. Female patients with irritable bowel syndrome and healthy controls underwent a battery of neuropsychological tests after a placebo or acute tryptophan depletion. The results showed that acute tryptophan depletion produces decreased hippocampal-mediated cognitive performance [67]. A similar test in female patients with diarrhea-predominant irritable bowel syndrome and healthy controls showed acute tryptophan depletion was significantly associated with impaired immediate and delayed recall performance in an affective memory test, though there was no difference in scores between patient and control groups [121]. These patients also showed an enhanced visceral perception to an aversive visceral stimulus during acute tryptophan depletion similar to the study by Labus et al [120].

Interestingly, acute tryptophan depletion has not been shown to have an effect on mucosal concentrations of serotonin or the metabolite 5-hydroxyindoleacetic acid [122]. However, acute tryptophan depletion studies investigating effects on regulation of gastrointestinal motility and sensation have shown lowered plasma tryptophan decreased the sensation of nausea during balloon distension without affecting gastric sensitivity and compliance. Acute tryptophan depletion also enhanced the postprandial intragastric volume increase, but this was not reflected by an increased nutrient intake [123]. In contrast, motor function of the rectum during acute tryptophan depletion was tested in female patients with diarrhea-predominant irritable bowel syndrome [124]. While the patient group had significantly altered rectal motor function, acute tryptophan depletion did not alter this.

Significant associations of tryptophan hydroxylase 1 gene polymorphisms, which may modify levels of circulating serotonin, are observed with irritable bowel syndrome-related cognitions in female patients. Employing the Cognitive Scale for Functional Bowel Disorders, tryptophan hydroxylase 1 gene polymorphisms were associated with negative cognitions regarding pain and anxiety around bowel movement. These polymorphisms were also associated with reductions in quality of life scores, in particular mental health and energy subscales, suggesting that subsets of the tryptophan hydroxylase 1 gene may impact the onset and course of irritable bowel syndrome, along with symptom severity and the emotional consequences of living with this disorder [125].

## 11. Concluding Remarks

As we have outlined in this review, experimental manipulation of tryptophan levels has allowed us to understand the role of central serotonin in mood and cognition. Low serotonin contributes to a lowered mood state, however this should be in concert with a biological or genetic manipulation, producing a predisposition that interacts with lowered serotonin to decrease mood. In addition, depleted serotonin causes cognitive impairments, with reports including deficits in verbal reasoning, episodic, and working memory, while conversely tryptophan supplementation has positive effects on attention and memory. Interestingly, emotional processing, the modification of memory that underlies emotion, is inhibited in subjects with depression, or has a high-risk to develop, after tryptophan depletion.

An influence of gut microbiota on behaviour is becoming increasingly evident, as is the extension to effects on tryptophan and serotonin metabolism. There is regulation of tryptophan and serotonin in the gut by the resident microbiota and recent studies show that low-to-no gut microbiota increases levels of tryptophan and serotonin and modifies central higher order behaviour.

Treatments for cognitive and mood disorders are an ongoing focus for neuroscience researchers and pharmaceutical organizations. The suggestion that the gut-microbiota has central influence opens up many new possibilities, especially with the suggestion from Mayer and colleagues [126] that the composition and metabolic activity of the gut microbiota may play a role in such brain disorders as autism, anxiety, and depression. Ongoing studies will, in time, evaluate these assertions and hopefully determine the mechanisms by which the gut microbiota affect mood and cognition.

## References

[1] Friedman M., Levin C.E. (2012). Nutritional and medicinal aspects of d-amino acids. Amino Acids.

[2] Herrera C.P., Smith K., Atkinson F., Ruell P., Chow C.M., O’Connor H., Brand-Miller J. (2011). High-glycaemic index and -glycaemic load meals increase the availability of tryptophan in healthy volunteers. Br. J. Nutr..

[3] Young V.R., Hussein M.A., Murray E., Scrimshaw N.S. (1971). Plasma tryptophan response curve and its relation to tryptophan requirements in young adult men. J. Nutr..

[4] Richard D.M., Dawes M.A., Mathias C.W., Acheson A., Hill-Kapturczak N., Dougherty D.M. (2009). l-tryptophan: Basic metabolic functions, behavioral research and therapeutic indications. Int. J. Tryptophan Res. IJTR.

[5] Stone T.W., Darlington L.G. (2002). Endogenous kynurenines as targets for drug discovery and development. Nat. Rev. Drug Discov..

[6] Stone T.W., Darlington L.G. (2013). The kynurenine pathway as a therapeutic target in cognitive and neurodegenerative disorders. Br. J. Pharmacol..

[7] Crockett M.J., Clark L., Roiser J.P., Robinson O.J., Cools R., Chase H.W., den Ouden H., Apergis-Schoute A., Campbell-Meikeljohn D., Seymour B. (2012). Converging evidence for central 5-HT effects in acute tryptophan depletion. Mol. Psychiatr..

[8] Hughes M.M., Carballedo A., McLoughlin D.M., Amico F., Harkin A., Frodl T., Connor T.J. (2012). Tryptophan depletion in depressed patients occurs independent of kynurenine pathway activation. Brain Behav. Immun..

[9] Van Donkelaar E.L., Blokland A., Ferrington L., Kelly P.A., Steinbusch H.W., Prickaerts J. (2011). Mechanism of acute tryptophan depletion: Is it only serotonin?. Mol. Psychiatr..

[10] Sanchez C.L., van Swearingen A.E.D., Arrant A.E., Biskup C.S., Kuhn C.M., Zepf F.D. (2015). Simplified dietary acute tryptophan depletion: Effects of a novel amino acid mixture on the neurochemistry of C57BL/6J mice. Food Nutr. Res..

[11] Fernstrom J.D., Fernstrom M.H. (2006). Exercise, serum free tryptophan, and central fatigue. J. Nutr..

[12] Berger M., Gray J.A., Roth B.L. (2009). The expanded biology of serotonin. Ann. Rev. Med..

[13] Hoyer D., Clarke D.E., Fozard J.R., Hartig P.R., Martin G.R., Mylecharane E.J., Saxena P.R., Humphrey P.P. (1994). International union of pharmacology classification of receptors for 5-hydroxytryptamine (serotonin). Pharmacol. Rev..

[14] Lesch K.P., Waider J. (2012). Serotonin in the modulation of neural plasticity and networks: Implications for neurodevelopmental disorders. Neuron.

[15] Waraich P., Goldner E.M., Somers J.M., Hsu L. (2004). Prevalence and incidence studies of mood disorders: A systematic review of the literature. Can. J. Psychiatry.

[16] Cleare A., Pariante C.M., Young A.H., Anderson I.M., Christmas D., Cowen P.J., Dickens C., Ferrier I.N., Geddes J., Gilbody S. (2015). Evidence-based guidelines for treating depressive disorders with antidepressants: A revision of the 2008 British Association for Psychopharmacology guidelines. J. Psychopharmacol..

[17] Morrissette D.A., Stahl S.M. (2014). Modulating the serotonin system in the treatment of major depressive disorder. Cns Spectr..

[18] Mulinari S. (2012). Monoamine theories of depression: Historical impact on biomedical research. J. Hist. Neurosci..

[19] Melander H., Salmonson T., Abadie E., van Zwieten-Boot B. (2008). A regulatory apologia—A review of placebo-controlled studies in regulatory submissions of new-generation antidepressants. Eur. Neuropsychopharmacol..

[20] Walsh B.T., Seidman S.N., Sysko R., Gould M. (2002). Placebo response in studies of major depression: Variable, substantial, and growing. Jama.

[21] Hindmarch I. (2002). Beyond the monoamine hypothesis: Mechanisms, molecules and methods. Eur. Psychiatry.

[22] Owens M.J. (2004). Selectivity of antidepressants: From the monoamine hypothesis of depression to the SSRI revolution and beyond. J. Clin. Psychiatry.

[23] Young S.N. (2013). Acute tryptophan depletion in humans: A review of theoretical, practical and ethical aspects. J. Psychiatry Neurosci. JPN.

[24] Meneses A. (1999). 5-HT system and cognition. Neurosci. Biobehav. R..

[25] Buhot M.C., Martin S., Segu L. (2000). Role of serotonin in memory impairment. Ann. Med..

[26] Ogren S.O., Eriksson T.M., Elvander-Tottie E., D’Addario C., Ekstrom J.C., Svenningsson P., Meister B., Kehr J., Stiedl O. (2008). The role of 5-HT(1A) receptors in learning and memory. Behav. Brain Res..

[27] Sharma T., Mockler D. (1998). The cognitive efficacy of atypical antipsychotics in schizophrenia. J. Clin. Psychopharm..

[28] Bockaert J., Claeysen S., Compan V., Dumuis A. (2011). 5-HT4 receptors, a place in the sun: Act two. Curr. Opin. Pharmacol..

[29] Wolf H. (2000). Preclinical and clinical pharmacology of the 5-HT3 receptor antagonists. Scand. J. Rheumatol..

[30] Cowen P., Sherwood A.C. (2013). The role of serotonin in cognitive function: Evidence from recent studies and implications for understanding depression. J. Psychopharmacol..

[31] Shopsin B., Friedman E., Gershon S. (1976). Parachlorophenylalanine reversal of tranylcypromine effects in depressed patients. Arch. Gen. Psychiatry.

[32] Biskup C.S., Sanchez C.L., Arrant A., van Swearingen A.E., Kuhn C., Zepf F.D. (2012). Effects of acute tryptophan depletion on brain serotonin function and concentrations of dopamine and norepinephrine in C57BL/6J and BALB/cJ mice. PLoS ONE.

[33] Ardis T.C., Cahir M., Elliott J.J., Bell R., Reynolds G.P., Cooper S.J. (2009). Effect of acute tryptophan depletion on noradrenaline and dopamine in the rat brain. J. Psychopharmacol..

[34] Jans L.A.W., Korte-Bouws G.A.H., Korte S.M., Blokland A. (2010). The effects of acute tryptophan depletion on affective behaviour and cognition in brown norway and sprague dawley rats. J. Psychopharmacol..

[35] Lieben C.K., Blokland A., Westerink B., Deutz N.E. (2004). Acute tryptophan and serotonin depletion using an optimized tryptophan-free protein-carbohydrate mixture in the adult rat. Neurochem. Int..

[36] Young S.N., Ervin F.R., Pihl R.O., Finn P. (1989). Biochemical aspects of tryptophan depletion in primates. Psychopharmacology.

[37] Young S.N., Smith S.E., Pihl R.O., Ervin F.R. (1985). Tryptophan depletion causes a rapid lowering of mood in normal males. Psychopharmacology.

[38] Riedel W.J., Klaassen T., Deutz N.E., van Someren A., van Praag H.M. (1999). Tryptophan depletion in normal volunteers produces selective impairment in memory consolidation. Psychopharmacology.

[39] Cahir M., Ardis T., Reynolds G.P., Cooper S.J. (2007). Acute and chronic tryptophan depletion differentially regulate central 5-HT 1A and 5-HT 2A receptor binding in the rat. Psychopharmacology.

[40] Nishizawa S., Benkelfat C., Young S.N., Leyton M., Mzengeza S., de Montigny C., Blier P., Diksic M. (1997). Differences between males and females in rates of serotonin synthesis in human brain. Proc. Natl. Acad. Sci. USA.

[41] Williams W.A., Shoaf S.E., Hommer D., Rawlings R., Linnoila M. (1999). Effects of acute tryptophan depletion on plasma and cerebrospinal fluid tryptophan and 5-hydroxyindoleacetic acid in normal volunteers. J. Neurochem..

[42] Moreno F.A., Parkinson D., Palmer C., Castro W.L., Misiaszek J., el Khoury A., Mathe A.A., Wright R., Delgado P.L. (2010). CSF neurochemicals during tryptophan depletion in individuals with remitted depression and healthy controls. Eur. Neuropsychopharmacol..

[43] Young S.N., Leyton M. (2002). The role of serotonin in human mood and social interaction. Insight from altered tryptophan levels. Pharmacol. Biochem. Behav..

[44] Toker L., Amar S., Bersudsky Y., Benjamin J., Klein E., Agam G. (2010). The biology of tryptophan depletion and mood disorders. Israel J. Psychiatry Relat. Sci..

[45] Mace J.L., Porter R.J., Dalrymple-Alford J.C., Wesnes K.A., Anderson T.J. (2011). The effects of acute tryptophan depletion on neuropsychological function, mood and movement in the healthy elderly. J. Psychopharmacol..

[46] Hughes J.H., Gallagher P., Stewart M.E., Matthews D., Kelly T.P., Young A.H. (2003). The effects of acute tryptophan depletion on neuropsychological function. J. Psychopharmacol..

[47] Ellenbogen M.A., Young S.N., Dean P., Palmour R.M., Benkelfat C. (1996). Mood response to acute tryptophan depletion in healthy volunteers: Sex differences and temporal stability. Neuropsychopharmacology.

[48] Feder A., Skipper J., Blair J.R., Buchholz K., Mathew S.J., Schwarz M., Doucette J.T., Alonso A., Collins K.A., Neumeister A. (2011). Tryptophan depletion and emotional processing in healthy volunteers at high risk for depression. Biol. Psychiatry.

[49] Van der Veen F.M., Evers E.A.T., Deutz N.E.P., Schmitt J.A.J. (2007). Effects of acute tryptophan depletion on mood and facial emotion perception related brain activation and performance in healthy women with and without a family history of depression. Neuropsychopharmacology.

[50] Smith K.A., Fairburn C.G., Cowen P.J. (1997). Relapse of depression after rapid depletion of tryptophan. Lancet.

[51] Moreno F.A., Gelenberg A.J., Heninger G.R., Potter R.L., McKnight K.M., Allen J., Phillips A.P., Delgado P.L. (1999). Tryptophan depletion and depressive vulnerability. Biol. Psychiatry.

[52] Booij L., van der Does A.J.W., Haffmans P.M.J., Riedel W.J., Fekkes D., Blom M.J.B. (2005). The effects of high-dose and low-dose tryptophan depletion on mood and cognitive functions of remitted depressed patients. J. Psychopharmacol..

[53] Booij L., van der Does A.J., Haffmans P.M., Riedel W.J. (2005). Acute tryptophan depletion in depressed patients treated with a selective serotonin-noradrenalin reuptake inhibitor: Augmentation of antidepressant response?. J. Affect. Disord..

[54] Delgado P.L., Price L.H., Miller H.L., Salomon R.M., Licinio J., Krystal J.H., Heninger G.R., Charney D.S. (1991). Rapid serotonin depletion as a provocative challenge test for patients with major depression: Relevance to antidepressant action and the neurobiology of depression. Psychopharmacol. Bull..

[55] Zhang L.M., Guadarrama L., Corona-Morales A.A., Vega-Gonzalez A., Rocha L., Escobar A. (2006). Rats subjected to extended l-tryptophan restriction during early postnatal stage exhibit anxious-depressive features and structural changes. J. Neuropathol. Exp. Neur..

[56] Uchida S., Kitamoto A., Umeeda H., Nakagawa N., Masushige S., Kida S. (2005). Chronic reduction in dietary tryptophan leads to changes in the emotional response to stress in mice. J. Nutr. Sci. Vitaminol..

[57] van Donkelaar E.L., Blokland A., Lieben C.K.J., Kenis G., Ferrington L., Kelly P.A.T., Steinbusch H.W.M., Prickaerts J. (2010). Acute tryptophan depletion in C57BL/6 mice does not induce central serotonin reduction or affective behavioural changes. Neurochem. Int..

[58] Mendelsohn D., Riedel W.J., Sambeth A. (2009). Effects of acute tryptophan depletion on memory, attention and executive functions: A systematic review. Neurosci. Biobehav. R..

[59] Schmitt J.A., Jorissen B.L., Sobczak S., van Boxtel M.P., Hogervorst E., Deutz N.E., Riedel W.J. (2000). Tryptophan depletion impairs memory consolidation but improves focussed attention in healthy young volunteers. J. Psychopharmacol..

[60] Booij L., van der Does A.J.W. (2011). Emotional processing as a predictor of symptom change: An acute tryptophan depletion study in depressed patients. Eur. Neuropsychopharm..

[61] Beacher F.D.C.C., Gray M.A., Minati L., Whale R., Harrison N.A., Critchley H.D. (2011). Acute tryptophan depletion attenuates conscious appraisal of social emotional signals in healthy female volunteers. Psychopharmacology.

[62] Epperson C.N., Amin Z., Ruparel K., Gur R., Loughead J. (2012). Interactive effects of estrogen and serotonin on brain activation during working memory and affective processing in menopausal women. Psychoneuroendocrino.

[63] Mace J.L., Porter R.J., Dalrymple-Alford J.C., Wesnes K.A., Anderson T.J. (2010). Effects of acute tryptophan depletion on neuropsychological and motor function in parkinson’s disease. J. Psychopharmacol..

[64] Zepf F.D., Landgraf M., Biskup C.S., Dahmen B., Poustka F., Wockel L., Stadler C. (2013). No effect of acute tryptophan depletion on verbal declarative memory in young persons with adhd. Acta Psychiatr. Scand..

[65] Crean J., Richards J.B., de Wit H. (2002). Effect of tryptophan depletion on impulsive behavior in men with or without a family history of alcoholism. Behav. Brain Res..

[66] Porter R.J., Lunn B.S., O’Brien J.T. (2003). Effects of acute tryptophan depletion on cognitive function in Alzheimer’s disease and in the healthy elderly. Psychol. Med..

[67] Kennedy P.J., Clarke G., O’Neill A., Groeger J.A., Quigley E.M., Shanahan F., Cryan J.F., Dinan T.G. (2014). Cognitive performance in irritable bowel syndrome: Evidence of a stress-related impairment in visuospatial memory. Psychol. Med..

[68] Kennedy P.J., Allen A.P., O’Neill A., Quigley E.M., Cryan J.F., Dinan T.G., Clarke G. (2015). Acute tryptophan depletion reduces kynurenine levels: Implications for treatment of impaired visuospatial memory performance in irritable bowel syndrome. Psychopharmacology.

[69] Von Ah D., Skaar T., Unverzagt F., Yu M.G., Wu J.W., Schneider B., Storniolo A.M., Moser L., Ryker K., Milata J. (2012). Evaluating the role of serotonin on neuropsychological function after breast cancer using acute tryptophan depletion. Biol. Res. Nurs..

[70] Blokland A., Lieben C., Deutz N.E.P. (2002). Anxiogenic and depressive-like effects, but no cognitive deficits, after repeated moderate tryptophan depletion in the rat. J. Psychopharmacol..

[71] Lieben C.K., van Oorsouw K., Deutz N.E., Blokland A. (2004). Acute tryptophan depletion induced by a gelatin-based mixture impairs object memory but not affective behavior and spatial learning in the rat. Behav. Brain Res..

[72] Rutten K., Lieben C., Smits L., Blokland A. (2007). The PDE4 inhibitor rolipram reverses object memory impairment induced by acute tryptophan depletion in the rat. Psychopharmacology.

[73] Van Donkelaar E.L., Rutten K., Blokland A., Akkerman S., Steinbusch H.W., Prickaerts J. (2008). Phosphodiesterase 2 and 5 inhibition attenuates the object memory deficit induced by acute tryptophan depletion. Eur. J. Pharmacol..

[74] Liu H.P., Zhou J., Fang L., Liu Z., Fan S.H., Xie P. (2013). Acute tryptophan depletion reduces nitric oxide synthase in the rat hippocampus. Neurochem. Res..

[75] Jenkins T.A., Elliott J.J., Ardis T.C., Cahir M., Reynolds G.P., Bell R., Cooper S.J. (2010). Tryptophan depletion impairs object-recognition memory in the rat: Reversal by risperidone. Behav. Brain Res..

[76] Uchida S., Umeeda H., Kitamoto A., Masushige S., Kida S. (2007). Chronic reduction in dietary tryptophan leads to a selective impairment of contextual fear memory in mice. Brain Res..

[77] Bortolato M., Frau R., Orru M., Collu M., Mereu G., Carta M., Fadda F., Stancampiano R. (2008). Effects of tryptophan deficiency on prepulse inhibition of the acoustic startle in rats. Psychopharmacology.

[78] Carta M., Fadda F., Stancampiano R. (2006). Tryptophan-deficient diet increases the neurochemical and behavioral response to amphetamine. Brain Res..

[79] Mohajeri M.H., Wittwer J., Vargas K., Hogan E., Holmes A., Rogers P.J., Goralczyk R., Gibson E.L. (2015). Chronic treatment with a tryptophan-rich protein hydrolysate improves emotional processing, mental energy levels and reaction time in middle-aged women. Br. J. Nutr..

[80] Booij L., Merens W., Markus C.R., van der Does A.J.W. (2006). Diet rich in alpha-lactalbumin improves memory in unmedicated recovered depressed patients and matched controls. J. Psychopharmacol..

[81] Murphy S.E., Longhitano C., Ayres R.E., Cowen P.J., Harmer C.J. (2006). Tryptophan supplementation induces a positive bias in the processing of emotional material in healthy female volunteers. Psychopharmacology.

[82] Rondanelli M., Opizzi A., Faliva M., Mozzoni M., Antoniello N., Cazzola R., Savare R., Cerutti R., Grossi E., Cestaro B. (2012). Effects of a diet integration with an oily emulsion of DHA-phospholipids containing melatonin and tryptophan in elderly patients suffering from mild cognitive impairment. Nutr. Neurosci..

[83] Hartmann E. (1982). Effects of l-tryptophan on sleepiness and on sleep. J. Psychiatr. Res..

[84] Silber B.Y., Schmitt J.A. (2010). Effects of tryptophan loading on human cognition, mood, and sleep. Neurosci. Biobehav. Rev..

[85] Markus C.R., Jonkman L.M., Lammers J.H., Deutz N.E., Messer M.H., Rigtering N. (2005). Evening intake of alpha-lactalbumin increases plasma tryptophan availability and improves morning alertness and brain measures of attention. Am. J. Clin. Nutr..

[86] Bhatti T., Gillin J.C., Seifritz E., Moore P., Clark C., Golshan S., Stahl S., Rapaport M., Kelsoe J. (1998). Effects of a tryptophan-free amino acid drink challenge on normal human sleep electroencephalogram and mood. Biol. Psychiatry.

[87] Carhart-Harris R.L., Nutt D.J., Munafo M.R., Christmas D.M., Wilson S.J. (2009). Equivalent effects of acute tryptophan depletion on rem sleep in ecstasy users and controls. Psychopharmacology.

[88] Nakamaru-Ogiso E., Miyamoto H., Hamada K., Tsukada K., Takai K. (2012). Novel biochemical manipulation of brain serotonin reveals a role of serotonin in the circadian rhythm of sleep-wake cycles. Eur. J. Neurosci..

[89] Tsuno N., Besset A., Ritchie K. (2005). Sleep and depression. J. Clin. Psychiatry.

[90] Beasley C.M., Sayler M.E., Weiss A.M., Potvin J.H. (1992). Fluoxetine-activating and sedating effects at multiple fixed doses. J. Clin. Psychopharm..

[91] Moore P., Gillin J.C., Bhatti T., DeModena A., Seifritz E., Clark C., Stahl S., Rapaport M., Kelsoe J. (1998). Rapid tryptophan depletion, sleep electroencephalogram, and mood in men with remitted depression on serotonin reuptake inhibitors. Arch. Gen. Psychiatry.

[92] Landolt H.P., Kelsoe J.R., Rapaport M.H., Gillin J.C. (2003). Rapid tryptophan depletion reverses phenelzine-induced suppression of rem sleep. J. Sleep Res..

[93] Voderholzer U., Riemann D., Huwig-Poppe C., Kuelz A.K., Kordon A., Bruestle K., Berger M., Hohagen F. (2007). Sleep in obsessive compulsive disorder-polysomnographic studies under baseline conditions and after experimentally induced serotonin deficiency. Eur. Arch. Psychiatry Clin. Neurosci..

[94] Sansone R.A., Sansone L.A. (2012). SSRIs: Bad to the bone?. Innov. Clin. Neurosci..

[95] Crane J.D., Palanivel R., Mottillo E.P., Bujak A.L., Wang H., Ford R.J., Collins A., Blumer R.M., Fullerton M.D., Yabut J.M. (2015). Inhibiting peripheral serotonin synthesis reduces obesity and metabolic dysfunction by promoting brown adipose tissue thermogenesis. Nat. Med..

[96] Gershon M.D. (1999). Review article: Roles played by 5-hydroxytryptamine in the physiology of the bowel. Aliment. Pharm. Ther..

[97] Amireault P., Sibon D., Cote F. (2013). Life without peripheral serotonin: Insights from tryptophan hydroxylase 1 knockout mice reveal the existence of paracrine/autocrine serotonergic networks. ACS Chem. Neurosci..

[98] Sakakibara Y., Katoh M., Kawayanagi T., Nadai M. (2015). Species and tissue differences in serotonin glucuronidation. Xenobiotica.

[99] Jandhyala S.M., Talukdar R., Subramanyam C., Vuyyuru H., Sasikala M., Reddy D.N. (2015). Role of the normal gut microbiota. World J. Gastroenterol..

[100] La Rosa P.S., Warner B.B., Zhou Y., Weinstock G.M., Sodergren E., Hall-Moore C.M., Stevens H.J., Bennett W.E., Shaikh N., Linneman L.A. (2014). Patterned progression of bacterial populations in the premature infant gut. Proc. Natl. Acad. Sci. USA.

[101] O’Mahony S.M., Clarke G., Borre Y.E., Dinan T.G., Cryan J.F. (2015). Serotonin, tryptophan metabolism and the brain-gut-microbiome axis. Behav. Brain Res..

[102] Clarke G., Grenham S., Scully P., Fitzgerald P., Moloney R.D., Shanahan F., Dinan T.G., Cryan J.F. (2013). The microbiome-gut-brain axis during early life regulates the hippocampal serotonergic system in a sex-dependent manner. Mol. Psychiatry.

[103] Wikoff W.R., Anfora A.T., Liu J., Schultz P.G., Lesley S.A., Peters E.C., Siuzdak G. (2009). Metabolomics analysis reveals large effects of gut microflora on mammalian blood metabolites. Proc. Natl. Acad. Sci. USA.

[104] Diaz Heijtz R., Wang S., Anuar F., Qian Y., Bjorkholm B., Samuelsson A., Hibberd M.L., Forssberg H., Pettersson S. (2011). Normal gut microbiota modulates brain development and behavior. Proc. Natl. Acad. Sci. USA.

[105] Spiller R. (2008). Serotonin and GI clinical disorders. Neuropharmacology.

[106] Nakai A., Kumakura Y., Boivin M., Rosa P., Diksic M., D’Souza D., Kersey K. (2003). Sex differences of brain serotonin synthesis in patients with irritable bowel syndrome using alpha-[11c]methyl-l-tryptophan, positron emission tomography and statistical parametric mapping. Can. J. Gastroenterol..

[107] Brint E.K., MacSharry J., Fanning A., Shanahan F., Quigley E.M.M. (2011). Differential expression of toll-like receptors in patients with irritable bowel syndrome. Am. J. Gastroenterol..

[108] McKernan D.P., Gaszner G., Quigley E.M., Cryan J.F., Dinan T.G. (2011). Altered peripheral toll-like receptor responses in the irritable bowel syndrome. Aliment. Pharm. Ther..

[109] Reigstad C.S., Salmonson C.E., Rainey J.F., Szurszewski J.H., Linden D.R., Sonnenburg J.L., Farrugia G., Kashyap P.C. (2015). Gut microbes promote colonic serotonin production through an effect of short-chain fatty acids on enterochromaffin cells. FASEB J..

[110] Neufeld K.M., Kang N., Bienenstock J., Foster J.A. (2011). Reduced anxiety-like behavior and central neurochemical change in germ-free mice. Neurogastroenterol. Motil..

[111] Bravo J.A., Forsythe P., Chew M.V., Escaravage E., Savignac H.M., Dinan T.G., Bienenstock J., Cryan J.F. (2011). Ingestion of lactobacillus strain regulates emotional behavior and central gaba receptor expression in a mouse via the vagus nerve. Proc. Natl. Acad. Sci. USA.

[112] O’Mahony S.M., Marchesi J.R., Scully P., Codling C., Ceolho A.M., Quigley E.M.M., Cryan J.F., Dinan T.G. (2009). Early life stress alters behavior, immunity, and microbiota in rats: Implications for irritable bowel syndrome and psychiatric illnesses. Biol. Psychiatry.

[113] Park A.J., Collins J., Blennerhassett P.A., Ghia J.E., Verdu E.F., Bercik P., Collins S.M. (2013). Altered colonic function and microbiota profile in a mouse model of chronic depression. Neurogastroenterol. Motil..

[114] Jiang H.Y., Ling Z.X., Zhang Y.H., Mao H.J., Ma Z.P., Yin Y., Wang W.H., Tang W.X., Tan Z.L., Shi J.F. (2015). Altered fecal microbiota composition in patients with major depressive disorder. Brain Behav. Immun..

[115] Gareau M.G., Wine E., Rodrigues D.M., Cho J.H., Whary M.T., Philpott D.J., MacQueen G., Sherman P.M. (2011). Bacterial infection causes stress-induced memory dysfunction in mice. Gut.

[116] Desbonnet L., Clarke G., Shanahan F., Dinan T.G., Cryan J.F. (2014). Microbiota is essential for social development in the mouse. Mol. Psychiatry.

[117] Bekkering P., Jafri I., van Overveld F.J., Rijkers G.T. (2013). The intricate association between gut microbiota and development of type 1, type 2 and type 3 diabetes. Expert Rev. Clin. Immun..

[118] Naseer M.I., Bibi F., Alqahtani M.H., Chaudhary A.G., Azhar E.I., Kamal M.A., Yasir M. (2014). Role of gut microbiota in obesity, type 2 diabetes and Alzheimer’s disease. CNS Neurol. Disord. Drug.

[119] Labus J.S., Mayer E.A., Jarcho J., Kilpatrick L.A., Kilkens T.O.C., Evers E.A.T., Backes W.H., Brummer R.J.M., van Nieuwenhoven M.A. (2011). Acute tryptophan depletion alters the effective connectivity of emotional arousal circuitry during visceral stimuli in healthy women. Gut.

[120] Kim H.M. (2012). Acute tryptophan depletion and functional brain imaging in irritable bowel syndrome. J. Neurogastroenterol..

[121] Kilkens T.O., Honig A., van Nieuwenhoven M.A., Riedel W.J., Brummer R.J. (2004). Acute tryptophan depletion affects brain-gut responses in irritable bowel syndrome patients and controls. Gut.

[122] Keszthelyi D., Troost F.J., Jonkers D.M., van Donkelaar E.L., Dekker J., Buurman W.A., Masclee A.A. (2012). Does acute tryptophan depletion affect peripheral serotonin metabolism in the intestine?. Am. J. Clin. Nutr..

[123] Geeraerts B., van Oudenhove L., Boesmans W., Vos R., Vanden Berghe P., Tack J. (2011). Influence of acute tryptophan depletion on gastric sensorimotor function in humans. Am. J. Physiol..

[124] Van Nieuwenhoven M.A., Kilkens T.O. (2012). The effect of acute serotonergic modulation on rectal motor function in diarrhea-predominant irritable bowel syndrome and healthy controls. Eur. J. Gastroenterol. Hepatol..

[125] Jun S.E., Kohen R., Cain K.C., Jarrett M.E., Heitkemper M.M. (2014). Tph gene polymorphisms are associated with disease perception and quality of life in women with irritable bowel syndrome. Biol. Res. Nurs..

[126] Mayer E.A., Knight R., Mazmanian S.K., Cryan J.F., Tillisch K. (2014). Gut microbes and the brain: Paradigm shift in neuroscience. J. Neurosci..

